# Pharmacokinetic modelling of intravenous immunoglobulin in children with primary immunodeficiencies and secondary antibody deficiencies

**DOI:** 10.1002/bcp.70420

**Published:** 2025-12-28

**Authors:** Iek Leng Cheng, Zhong Hui Huang, Austen Worth, Claire Booth, Joseph F. Standing

**Affiliations:** ^1^ Infection, Immunity and Inflammation Research & Teaching Department, UCL GOS Institute of Child Health University of London London UK; ^2^ Pharmacy Department Great Ormond Street Hospital London Greater London UK; ^3^ Immunology Department Great Ormond Street Hospital London Greater London UK

**Keywords:** children, chimeric antigen receptor T cell therapy, immunoglobulin, pharmacokinetic modelling, primary immunodeficiency, rituximab, secondary antibody deficiency

## Abstract

**Aims:**

Children with primary immunodeficiency (PID) and secondary antibody deficiency (SAD) often require immunoglobulin replacement therapy due to low plasma immunoglobulin G (IgG) levels and recurrent infections. Existing pharmacokinetic models for immunoglobulin in PID patients predominantly focus on adults, with limited attention to secondary antibody deficiencies and a lesser emphasis on paediatric populations. This study aims to investigate the pharmacokinetic properties of IgG in paediatric patients with PID and SAD.

**Methods:**

Population pharmacokinetic analysis for PID and SAD children treated with intravenous immunoglobulin at a tertiary paediatric centre was conducted using NONMEM® (7.5.1). Dosing simulations to achieve therapeutic levels of 6 and 8 gL^−1^ were performed.

**Results:**

A population pharmacokinetic analysis of 64 patients (median age 4.08 years, range 0.06–16.8) was performed. A two‐compartment model with first‐order elimination, incorporating both additive and proportional residual error, adequately described the data. Interindividual variability was modelled on clearance, volume of distribution and baseline IgG levels, with allometric scaling to a 70‐kg body weight applied a priori. The estimated clearance was 0.308 L^−1^ day^−1^ 70 kg^−1^ (95% CI 0.23, 0.67), and the volume of distribution was 10.96 L^−1^ 70 kg^−1^ (95% CI 5.97, 15.79). Patients with SAD exhibited a lower clearance rate of 54% compared with PID patients. Dosing simulations indicated that the recommended SAD dosing regimen maintained therapeutic IgG levels in the simulated population. However, only 44.8% to 51.9% of patients with PID achieved target IgG levels with the standard regimen.

**Conclusions:**

This study provides insights into immunoglobulin pharmacokinetics in paediatric PID and SAD patients, guiding optimised dosing strategies. Administering a loading dose would improve the probability of maintaining therapeutic IgG levels during the 4‐week dosing interval.

**What is already known about this subject**

- Existing pharmacokinetic models for immunoglobulin in primary immunodeficiency patients predominantly focus on adults, with limited attention to secondary antibody deficiencies and a lesser emphasis on pediatric populations.
- The growing use of B cell‐depleting therapies (e.g., CAR‐T cell therapy, rituximab) and newer monoclonal antibodies therapies impacting on B cell function, combined with improved diagnosis of primary immunodeficiency disorders, increase the discrepancy between global supply and demand.

**What this study adds**

- Secondary antibody deficiency patients exhibit 54% lower clearance than primary immunodeficient patients meaning for the same dose, they will have higher immunoglobulin concentrations.
- Primary immunodeficiency patients are less likely to achieve therapeutic targets with the recommended dosing regimen. A loading dose could improve the probability of maintaining therapeutic levels.

## 1 INTRODUCTION

Immunoglobulin replacement therapy (IgRT) is used to manage patients with both primary immunodeficiency (PID) and secondary antibody deficiency (SAD). In contrast to PID, which results from genetic defects of the immune system, SAD arises from external influences such as malnutrition, HIV infection and haematological malignancies and their treatment.^1^, ^2^, ^3^, ^4^ Recent years have seen the introduction of novel therapies targeting B cells, such as rituximab and chimeric antigen receptor T cell (CAR‐T) therapy, which are now available for children to treat autoimmune diseases and haematological conditions.^5^, ^6^ Increased use of B cell‐depleting novel therapies, as well as improved recognition and diagnosis of PID disorders, contributed to a 6% to 8% annual increase in the demand for immunoglobulin G (IgG) globally between 2010 and 2018.^7^ Yet, with the significant geographical imbalance in the global supply of plasma and the cost burden, the use of IgG needs to be rationalized.

Children with PID and SAD can have serious, prolonged and sometimes life‐threatening infections.^8^, ^9^ PID patients with severely impaired or absent humoral response are managed by a combination of prophylactic antimicrobials and IgRT. Immunoglobulins, produced by B cells in response to antigens, are crucial for adaptive immunity, with IgG being the most abundant in peripheral blood, and vital against extracellular infections. Despite its routine use, there is a paucity of published evidence on the management of IgRT in PID children. Guideline‐recommended starting doses for PID patients range between 0.4–0.6 g kg^−1^ per month, with target troughs recommended at ≥8–10 g L^−1^ by British Society for Immunology and United Kingdom Primary Immunodeficiency Network (BSI‐UKPIN), or >5–8 g L^−1^ by American Academy of Allergy Asthma and Immunology (AAAI), all with dose adjustment based on clinical response.^10^, ^11^, ^12^ Rituximab and CAR‐T cells are engineered to target different antigens on B cells, resulting in B cell aplasia. B cell suppression typically lasts for 6 months but can persist for years with profound effects.^13^, ^14^ Hypogammaglobulinaemia is more frequent in children post CAR‐T therapy compared with adults, with increased severity and duration of hypogammaglobulinaemia.^14^, ^15^ IgRT in SAD is less well established.^16^ Due to the lack of clinical trials and robust studies to support its use, dosing is based on experiences from PID.^17^ The current recommended dose for SAD is 0.4–0.6 g kg^−1^ every 4 weeks, with dose adjustments based on clinical response.^16^, ^18^ The optimal target trough level is unclear and experts have not reached a consensus.^10^, ^12^, ^17^, ^18^ Overall, the target trough IgG levels have been suggested to remain above 6 g L^−1^,^10^, ^12^ with some patients with severe disease burden potentially benefitting from a trough level of 10 g L^−1^.^10^, ^17^ For the pediatric cohort, recommendations on the target trough level are even more limited. NHS England suggests keeping at least the lower limit of the age‐specific serum IgG reference range.^12^

Pharmacokinetic Ig studies that include pediatric cohorts are summarized in Table 1. The majority of the patient cohorts in these studies consist of patients with PID. Evaluated covariates include disease type and severity, age, total body weight, concurrent use of immunosuppressants, gender and comorbidities. Incorporating weight as a covariate consistently improved the model performance across these studies. The measured IgG plasma levels reflect both endogenous and exogenous IgG, complicating the assessment of the specific effects of exogenous IgG following an infusion. To isolate the exogenous IgG component, most studies assumed a fixed endogenous IgG level of 4 g L^−1^. However, Fokkink et al.^29^ and Li et al.^26^ successfully estimated the endogenous baseline IgG level as a model parameter. The presence of B cells, which produce IgG, can be an indicator of endogenous IgG production. CD19+ B cell count is one of the most reliable surface biomarkers for B cells, as CD19 is expressed from the pre‐B cell stage through to terminal differentiation into plasma cells.^30^ Fokkink et al.^29^ tested the influence of CD19+ B cell count on the baseline IgG levels and it had a significant impact on model improvement. The measurement of IgM can also serve as an indicator of B cell function. Upon activation, B cells initially produce IgM during the early stages of a primary antibody response. As the immune response matures activated B cell clones undergo immunoglobulin class switching, changing predominant antibody production away from IgM to other more specialized antibody classes, including IgG.^31^ The inclusion of these B cell‐related biomarkers in pharmacokinetic models can enhance the accuracy of exogenous IgG level estimation.

**TABLE 1:** Published population pharmacokinetic model summary.

Author, year (ref)	Population characteristics			Sample size	Dose	Covariates tested	Covariate retained in final model	Endogenous Ig level (g L^−1^)	Pharmacokinetic parameter estimates	
	Patient cohort	Median/mean age, year (range)	Median weight, kg (range)						CL 70 kg^−1^ (L day^−1^ 70 kg^−1^) (CI)	V 70 kg ^−1^ (L 70 kg^−1^) (CI)
Landersdorfer et al., 2013^19^	PID	24.5 (3–81)	53.5–66.5	151		Total body weight	Total body weight on CL and Vc	4 (FIX)	0.155 (0.143, 0.165)	Vc = 4.45 (3.97, 5.26) Vp = 4.72 (3.83, 5.85)
Tortorici et al., 2019^20^	PID + SAD	PID: 29.8 (3–81) SID: 69.5 (21–84)	72	187	IV: 473 ± 133 mg kg^−1^, monthly (PID) IV: 216 ± 106 mg kg^−1^, monthly (SID)	Body weight, age, sex and disease type (PID or SID)	Body weight on CL and Vc and disease type on Vc	4 (FIX)	0.149 (0.137, 0.157)	PID: Vc = 2.99 (2.42, 3.55) SID: Vc = 8.50 (3.53, 14.90) Both: Vp = 1.75 (1.45, 2.04)
Dumas et al., 2019^21^	PID	32.1 (3–81)	63.7	81	IV: 300–1000 mg kg^−1^, 3–4 weekly SC: weekly equivalent doses	Age, body weight, geographic region, race and sex	Body weight on CL	9.9	0.0922 (not reported)	4.01 (not reported)
Zhang et al., 2020^22^	PID	23 (3–81)	66 (13–135)	173	IV: 451–460 mg kg^−1^, 3–4 weekly SC: 118.7–234 mg kg^−1^, weekly to biweekly	Body weight	Body weight on CL and Vc	4 (FIX)	0.144 (0.135, 0.154)	Vc = 4.05 (3.23, 4.86) Vp = 4.71 (3.12, 6.29)
Luo et al., 2020^23^	PID	21 (3–81)	58.7	202	IV: 268.5–472.9 mg kg^−1^, 3–4 weekly SC: 108.0–209.0, weekly to biweekly	Body weight, ethnicity, age and sex	Body weight on CL and Vc	4 (FIX)	0.159 (0.148, 0.170)	Vc = 4.79 (3.98, 5.72) Vp = 4.19 (2.66, 4.73)
Tegenge and Mahmood, 2020^24^	Very low birth weight neonates	Chronological age of neonates: 3.0 days (1–6)	1.09	20	Single IV low dose: 500 mg kg^−1^ Single IV high dose: 750 mg kg^−1^	Body weight	None	5 (FIX)	0.06656 (not reported)	Vc = 0.558 (not reported) Vp = 3.532 (not reported)
Lee et al., 2021^25^	PID	9.5 (3–64)	27	10	IV: 360–600 mg kg^−1^, 3–4 weekly	Age, total body weight, ethnicity, sex, presence of bronchiectasis and genotype	Weight	0.7 (median measured baseline Ig)	0.1261 (0.097, 0.1456)	Vc = 5.10 (4.38, 5.62)
Li et al., 2022^26^	PID	31.5 (2–83)	47.3	340	480 mg kg^−1^, 3–4 weekly	Age, body size, IgG product, presence of hyaluronidase and sex	Lean body weight	6.15 (estimated in model)	0.208 (0.190, 0.226)	Vc = 3.58 (3.39, 3.77) Vp = 1.66 (1.07, 2.26)
Navarro‐Mora et al., 2022^27^	PID	29 (2–72)	65.7	95	IV: 495 mg kg^−1^ (278–902) every 3–4 weeks SC: 184.8 (72.0–303.5)	SC formulation, sex, age and weight	Weight	4 (FIX)	0.157 (0.148, 0.167)	Vc = 3.19 (3.05, 3.34) Vp = 2.06 (1.57, 2.55)
Lee et al., 2024^28^	PID	15 (0.08–70)	43	79	IV: 360–600 mg kg^−1^, 3–4 weekly	Age, disease type, baseline IgG, comorbidity, ethnicity, genotype, sex and weight	Disease type and weight	9.08	0.0519 (0.0346, 0.0692)	Vc = 1.17 1.95 (1.04, 2.88) Vp = 0.85 (0.114, 1.77)

Abbreviations: CI, confidence interval; IV, intravenous; PID, primary immunodeficiency; SAD, secondary antibody deficiency; SC, subcutaneous; Vc, central volume of distribution; Vp, peripheral volume of distribution.

This study aimed to develop a pharmacokinetic model to inform dosing strategies for intravenous immunoglobulin replacement therapy in PID and SAD pediatric patients.

## 2 MATERIALS AND METHODS

### 2.1 Patient data

A retrospective analysis of de‐identified data captured by electronic health records in a tertiary pediatric hospital was performed. Children who received intravenous Ig for PID and SAD between April 2019 and April 2024 were included. Current dosing policy mirrors that of NHSE immunoglobulin commissioning policy,^12^ where PID patients receive 0.3 g kg^−1^ every 3 weeks and SAD patients receive 0.5 g kg^−1^ every 4 weeks. As the analysis utilized de‐identified retrospective data, patients' and parents' informed consent was not required. The study received approval from the London and Southeast Research Ethics Committee (Reference No. 21/LO/0646).

De‐identified data were collected, including demographics (age, weight and sex), treatment details (dose and timing of Ig), medical history (cause of SAD) and immunology blood results (baseline IgG levels, absolute CD19+ B cell count, IgM and IgG plasma levels). Plasma IgG levels were measured using an immunoturbidimetric assay, with a lower limit of quantification (LLOQ) of 0.07 g L^−1^.

### 2.2 Model development

The base structural model was first evaluated as one‐ and two‐compartment models. Residual variability was assessed according to an additive and/or proportional error model. Interindividual variability (IIV) was evaluated for clearance, volume of distribution and baseline IgG, assuming a log‐normal distribution. For the base model selection, the Akaike information criteria (AIC) was used to compare non‐nested models, while the objective function value (OFV) was utilized for nested model comparisons.

In this study, measured IgG was assumed to be the sum of endogenous IgG, the baseline IgG (CBAS) level prior to treatment and exogenous therapeutic Ig.^29^ Therefore, individual prediction of total IgG in the model incorporated both the baseline of Ig and the prediction from the model representing the contribution of the therapeutic drug.

The IgG levels prior to treatment initiation for children with SAD were assumed to be baseline Ig levels (median 3.11 g L^−1^). As all PID patients were established long‐term patients on IgRT, their baseline IgG levels were assumed to be 4 g L^−1^.^32^

Allometric size based on weight scaling of clearance and volume terms were added a priori:

(1) $$\text{Paramete}r_{i}=\text{Paramete}r_{\mathrm{pop}}\times {\left(\frac{c_{i}}{\bar{c}}\right)}^{\theta }\times \exp\left(\eta_{1}\right),$$

where *Parameter*_*pop*_ is the parameter estimates of population and *Parameter*_*i*_ is individual estimate of parameter. *c*_*i*_ is the individual value of the covariate and $\bar{c}$ is the typical value of the covariate in the population. In the fixed allometric weight scaling, *c*_*i*_ represented the individual body weight, with $\bar{c}$ set to 70 kg. The parameter *θ* assigned a power of 0.75 for clearance and intercompartmental clearance, while the volume of distribution was scaled with a power of 1.^33^ Stepwise forward inclusion and elimination were used to select covariates following the chi‐squared distribution; a drop in the log‐likelihood ratio of >3.84 per degree of freedom (*p* < .05), corresponding to each covariate being tested, was needed to be significant at a level of >6.63 per degree of freedom (*p* < .01). Tested covariates were selected based on known potential effects on Ig pharmacokinetics, including patient demographics (sex, weight and age) and laboratory data (absolute CD19+ B cell count and IgM) and type of antibody deficiency.

The effects of sex and antibody deficiency (PID *vs*. SAD) on clearance (CL) and volume of distribution (Vd) were explored. Sex and disease type were coded as binary categorical variables, with males and PID coded as 0 and females and SAD coded as 1. The effects of sex and disease type on CL and Vd were introduced into the model, both parameterized by *θ*_1_. The model equations for Cl and Vd are given by

(2) $$CL_{i}=CL_{\mathrm{pop}}\times \theta_{1}^{c_{i}}\times \exp\left(\eta_{1}\right),$$

and similarly for Vd, with corresponding population parameters.

The impact of age, absolute CD19+ B cell count and IgM levels on CL, Vd and CBAS were also evaluated. CBAS represents the typical baseline IgG concentration for the population, while absolute CD19+ B cell count *θ*_*CD*19_ and IgM level *θ*_*IgM*_ are individual‐specific values. The effect of age, CD19+ B cell count and IgM were evaluated as follows:

(3) $$CL_{i}=CL_{\mathrm{pop}}\times {\left(\frac{c_{i}}{\bar{c}}\right)}^{\theta_{\text{Covariate}}}\times \exp\left(\eta_{1}\right),$$

and similarly for Vd and CBAS, with corresponding population parameters where 4.08 is the median value of age (years), 0.8 for absolute CD19+ B cell count (× 10^−9^ cell L^−1^) and 0.21 is the median value of IgM level (g L^−1^).

Due to the presence of younger children (under 2 years old) in the population, postmenstrual age (PMA) was evaluated using the sigmoid hyperbolic or Hill's model^33^:

(4) $$CL_{i}=CL_{\mathrm{pop}}\times \frac{\mathrm{PM}A^{\text{Hill}}}{\mathrm{PM}A^{\text{Hill}}+\mathrm{PM}A_{50}^{\text{Hill}}},$$

where *PMA* is the postmenstrual age in weeks, *PMA*_*50*_ is the PMA when CL has reached 50% of adult function and *Hill* relates to the shape or sigmoidicity of clearance.

Population pharmacokinetic modelling and simulation were conducted using a first‐order conditional estimation method with interaction (FOCEI) in NONMEM (7.5.1). Graphical diagnostics developed with Xpose (4.7.2) guided model development. In conjunction with the information criterion, the goodness‐of‐fit plots (observation *vs*. population prediction [PRED], observation *vs*. individual prediction, conditional weighted residual [CWRES] *vs*. time, CWRES *vs*. PRED) and prediction‐corrected visual predictive checks, non‐parametric bootstrap (1000 samples) and realistic parameter estimates were used as criteria for model selection.

### 2.3 Dose simulations

Simulations were performed using the final population pharmacokinetic model in NONMEM (7.5.1) with parameter uncertainty incorporated via sampling from the variance–covariance matrix (*n* = 1000 replicates). A total of 1000 virtual pediatric patients were generated using the median values of relevant covariates (weight, IgM, type of immunodeficiency and absolute CD19+ B cell count) observed in the modelled cohort, with between‐subject and residual variability retained from the model. Simulated regimens included maintenance doses of 0.4, 0.5 and 0.6 g kg^−1^ every 21 or 28 days, with and without a single loading dose of 1 g kg^−1^ on Day 0. These regimens were selected to reflect clinically practical dosing used in PID and SAD patients. In line with NHS England commissioning policy, these patients minimal target level of PID was set to be >8 and 6 g L^−1^ for SAD. For each regimen, median and 95% prediction intervals for plasma IgG concentrations over the dosing interval were generated, and the proportion of days with concentrations above the lowest recommended therapeutic thresholds was calculated.

### 2.4 Nomenclature of targets and ligands

Key protein targets and ligands in this article are hyperlinked to corresponding entries in http://www.guidetopharmacology.org and are permanently archived in the Concise Guide to PHARMACOLOGY 2021/22.^34^, ^35^

## 3 RESULT

Sixty‐four children, aged between 3 weeks to 16.8 years, with a median weight of 18.6 (3.15–95.3 kg), received intravenous Ig for PID and SAD during the study period. Four hundred forty‐four blood samples were taken at various time points (Tables 2 and 3). The cohort consisted of 44 PID and 20 SAD patients, of which 15 of SAD patients had antibody deficiencies due to rituximab, and five due to CAR‐T cell therapy. The overall median baseline IgG level before the initiation of IgRT was 4 g L^−1^ (0.64–6.1 g L^−1^) and the absolute CD19+ B cell count was 0.07 × 10^−9^ cell L^−1^ (0–6.07 × 10^−9^ cell L^−1^). The median dose given was 0.56 g kg^−1^ (0.24 to 1.38 g kg^−1^) every 28 days. All observations were above the limit of quantification.

**TABLE 2:** Patient demographics.

Patient demographics	Total	PID	SAD
No. of patients	64	44	20
Age (years)	4.08 (0.06–16.8)	4.02 (0.06–16.8)	8.37 (0.6–16.2)
Median weight (kg)	18.6 (3.15–95.3)	13.65 (3.15–56.6)	22.1 (5.6–95.3)
Sex (M)	41	29	12
Median dose (g kg^−1^)	0.56 (0.24–1.38)	0.64 (0.24–1.38)	0.52 (0.27–1.09)
Baseline Ig (g L^−1^)	4 (0.64–6.1)	4 FIX	4 (0.64–4.92)
IgM (g L^−1^)	0.21 (0.03–5.61)	0.23 (0.03–4.97)	0.22 (0.05–5.61)
Absolute CD19+ cell count × 10^−9^ cell L^−1^	0.07 (0–6.07)	0.12 (0–6.07)	0.07 (0–0.85)

Abbreviations: PID, primary immunodeficiency; SAD, secondary antibody deficiency.

**TABLE 3:** Summary of IVIG products administered.

Products	Manufacturer	No. of patients	Median dose (g kg^−1^) (range)	No. of PID patients
Privigen	CSL Behring UK Limited	53	0.58 (0.27–1.38)	37
Octagam	Octapharma Limited	9	0.54 (0.24–1.09)	6
Gamunex	Grifols UK Ltd	2	0.36 (0.36–1.27)	1

### 3.1 Pharmacokinetic modelling

The pharmacokinetic data were adequately described by a two‐compartment model, which showed a significant reduction in the OFV by 115.0 and the AIC by 162.6 compared with the one‐compartment model. Goodness‐of‐fit plots also indicated a better fit to the observed data. IIV was parameterised using a variance–covariance matrix to quantify population variability for CL, Vd and CBAS. A combined error model (additive and proportional) was employed to describe residual variability. Allometric exponents for clearance (*θ*_CL_) and volume (*θ*_V_) were first estimated in the base model. The estimated *θ*_CL_ was 0.783 (SE 0.119; 95% CI 0.55, 1.02), and *θ*_V_ was 0.743 (SE 0.128; 95% CI 0.492, 0.994). Both confidence intervals included the theory‐based values of 0.75 (clearance) and 1.0 (volume).^33^ However, estimating *θ*_V_ was associated with a marked loss of precision in distribution volumes and intercompartmental clearance (e.g., V1 RSE increased from 10.7% to 30.5%), indicating parameter collinearity. When only *θ*_CL_ was estimated, the value (0.788; SE 0.109; 95% CI 0.574, 1.002) remained close to the theoretical 0.75 without improving model fit (ΔOFV = 0.17, not significant). Based on these results, the exponents of the final model were fixed. Typical parameter estimates for CL, intercompartmental clearance (*Q*) and Vd were allometrically scaled to a 70 kg individual. The estimated values were 0.308 L day^−1^ 70 kg^−1^ for CL and 10.96 L 70 kg^−1^ for Vd (Table 4). All typical parameter estimates had relative standard errors (RSEs) below 20%, and shrinkage for the random effects was no larger than 40%. The model demonstrated good robustness, as confirmed by bootstrap analysis, with bootstrap estimates closely matching the final parameter estimates.

**TABLE 4:** Pharmacokinetic parameter estimates for the base and final models including the bootstrap analysis.

Final model parameter estimates	Estimates (RSE%) [shrinkage%]	Bootstrap median	Bootstrap 95% confidence interval
CL (L day^−1^ 70 kg^−1^)	0.308 (15)	0.401	0.23–0.67
V1 (L 70 kg^−1^)	3.59 (15)	3.8	2.37–4.59
Q (L day^−1^ 70 kg^−1^)	1.08 (16)	1.09	0.91–5.44
V2 (L 70 kg^−1^)	7.37 (20)	6.97	3.6–11.2
CBAS (g L^−1^)	5.67 (10)	6.02	5.0–7.22
Type of immunodeficiency on CBAS	0.541 (18)	0.502	0.37–0.64
Type of immunodeficiency on CL	0.542 (21)	0.44	0.22–0.65
IgM on CBAS	0.11 (10)	0.1	0.03–0.16
IIV CL (%)	42.7 (27) [36]	47.83	28.5–75.9
IIV V2 (%)	138.6 (30) [35]	137.1	80.5–187.8
IIV CBAS (%)	49.3 (16) [13]	44.7	32.1–57.9
Additive error (g L^−1^)	0.812 (11) [11]	0.595	0.09728–0.9964
Proportional error (%)	11.7 (10%) [11]	2.27	0.786–7.68

*Note*: All parameters are allometrically scaled to 70 kg.

Incorporating antibody deficiency type on both CL and CBAS, as well as IgM cell count on CBAS, significantly improved the model's fit, with a reduction in the OFV by 52.5 and improved visual diagnostic plots. The difference in apparent clearance between SAD and PID patients resulted in a significant variation in clearance rates, with the clearance for SAD patients being half that of PID patients. In contrast, no significant decrease in OFV was found when introducing age, PMA, sex and absolute CD19+ cell count, hence, not significantly contribute to further improvement of the model.

## 4 SIMULATION RESULTS

**FIGURE 1:**
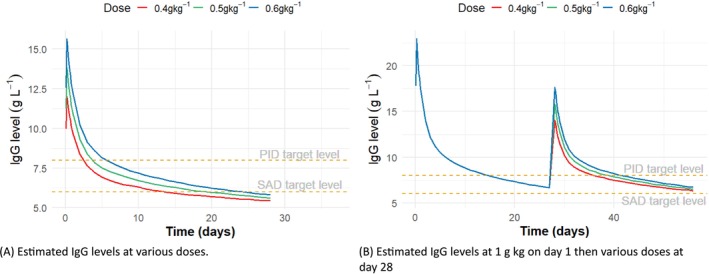
Estimated IgG levels at various dosing regimens. Dotted lines represent suggested therapeutic levels of 6 and 8 g L^−1^.

**FIGURE 2:**
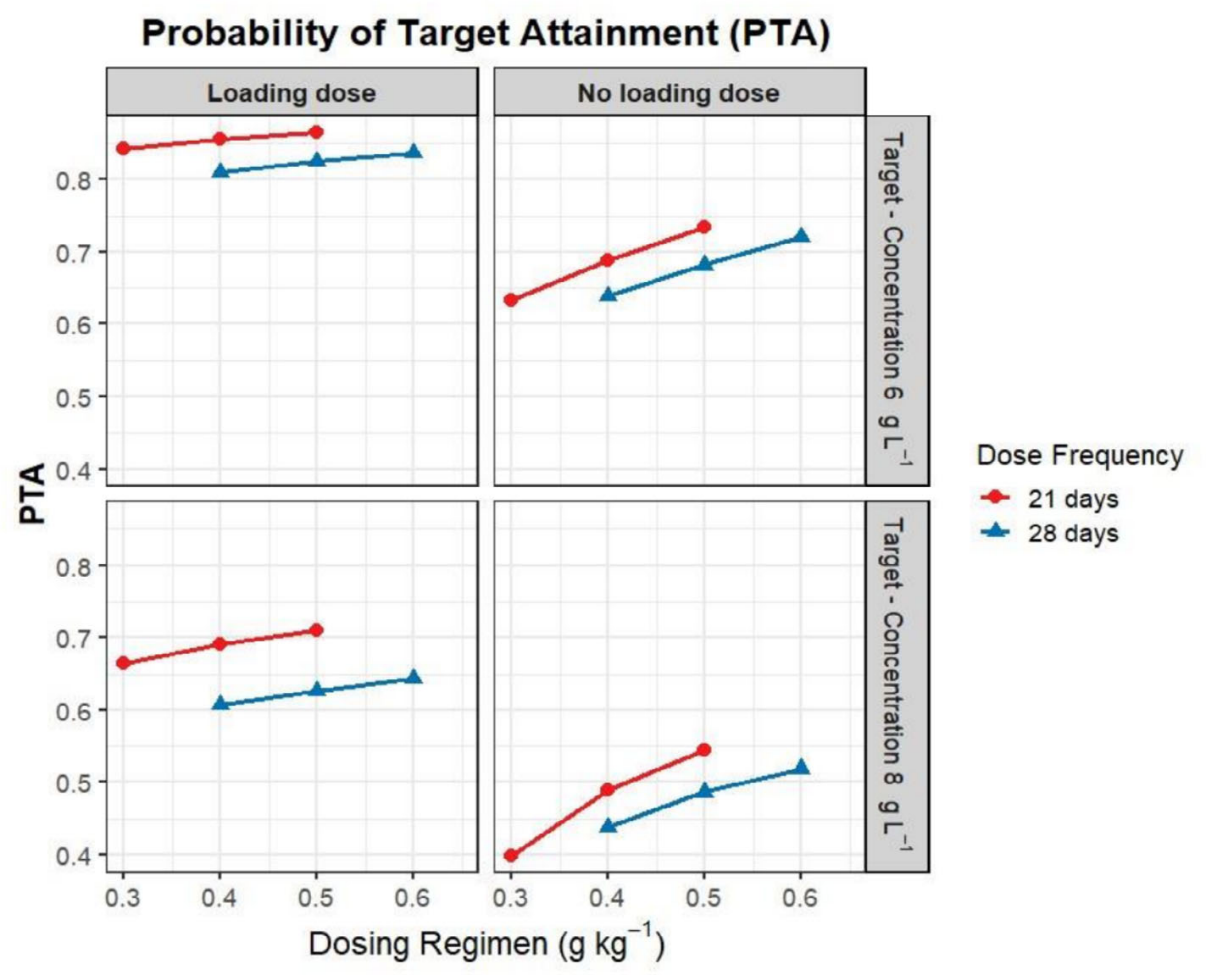
Estimated probability to achieve the recommended levels of 6 and 8 g L^−1^ with or without loading dose. Loading dose = 1 g kg^−1^ Day 0 then various maintenance doses at Day 21 or 28.

The median concentrations at the 50% percentile for each simulation time point were shown in Figure 1. Following infusions of 0.4, 0.5 and 0.6 g kg^−1^, the predicted exposure (area under the curve [AUC]) was 200.2 (CI 194.4, 206.2), 211.5 (CI 205.6, 217.5) and 222.4 (CI 216.6, 228.5) g L^−1^ day^−1^, respectively, over a 28‐day interval. While the probability of achieving the therapeutic target of SAD patients of 6 g L^−1^ was between 63.8% (CI 60.9, 66.6) and 72.1% (CI 69.4, 74.4), the likelihood of achieving the PID therapeutic target of 8 g L^−1^ decreased to 43.8% to 51.9%. The simulated plasma levels dropped below 8 g L^−1^ at 5, 6 and 7 days post infusion, respectively. Comparisons were made between a one‐off loading dose of 1 g kg^−1^ at Day 0 and no‐loading dose scenarios of the recommended doses to evaluate optimal dosing strategies. Following infusions of 0.5 g kg^−1^ with or without 1 g kg^−1^ on Day 0, the normalized AUC per day was 7.6 (CI 7.3, 7.8) and 17.9 (CI 17.5, 18.2) g L^−1^ day^−1^, respectively, allowing 48.7% (CI 46.0, 51.8) and 62.7% (CI 60.1, 65.2) of dosing intervals to maintain a concentration above 8 g L^−1^ (Figure 2). Reducing the dosing interval from 0.4 g kg^−1^ every 28 days to 0.3 g kg^−1^ every 21 days did not improve predicted exposure, yielding normalized AUC values of 7.2 (CI 7.20, 7.58) g L^−1^ day^−1^ and 6.9 (CI 6.75, 7.14)g L^−1^ day^−1^, respectively. However, when a 1 g kg^−1^ loading dose was administered at Day 0, the percentage of target attainment (PTA) increased with a shorter dosing interval.

## 5 DISCUSSION

This study elucidates the pharmacokinetic properties of intravenous Ig in pediatric patients with PID and SAD using real‐world clinical data collected from a diverse pediatric population. The pharmacokinetics were proficiently described by a two‐compartment model, allometrically scaled to 70 kg, incorporating IIV on CL, Vd and baseline Ig level. We evaluated a maturation function because age‐related maturation of IgG handling is biologically plausible: Neonatal B cells are largely naïve and class‐switching is limited,^36^ and although switched B cells and memory subsets gradually increase, they only reach adult‐like frequencies by adolescence and IgG levels reach only ~70% of adult concentrations by 1 year of age.^37^ As González‐Sales et al. note, reliable estimation of allometric exponents requires wide dispersion in the size metric (e.g., bodyweight) and sufficient data across developmental stages.^38^ While our dataset covered a substantial weight range (3.15–95.3 kg), the sample size was relatively small (*n* = 64) and entirely pediatric, with limited coverage of older adolescents and no adults. Under these conditions, simultaneous estimation of size exponents and a maturation function proved unstable and did not improve model fit or reduce unexplained variability. For these reasons, fixed theory‐based allometric exponents (CL ∝ WT^0.75^, V ∝ WT^1.0^) were applied. Some studies have questioned the use of theory‐based allometry in combination with maturation models, as this approach can risk over‐parameterisation or obscure the true maturation signal.^39^, ^40^ In our case, fixing the exponents allowed size effects to be standardized, reducing collinearity with age and enabling a cleaner evaluation of maturation. The absence of improvement in fit suggests that any maturation effect was either small relative to IIV or not sufficiently informed by the available data. A contributing factor may be the presence of maternally transferred IgG, which can sustain IgG levels in infants for up to 6 months post birth.^41^

The PID clearance (0.308 L day^−1^ 70 kg^−1^) is moderately higher than several adult leaning PID models (Table 1), which is plausible given residual maturation effects in children (even after theory‐based allometry), as well as trough‐enriched sampling and inter‐study assay/formulation differences. In contrast, the estimated Ig clearance in our pediatric SAD cohort was lower than in PID. At first glance, this conflicts with Tortorici et al., who reported similar CL in adult PID and SAD with a larger Vd in SAD.^20^ One explanation lies in cohort differences. In the United Kingdom, SAD patients usually initiate IgRT when baseline IgG is <4 g L^−1^,^12^ and in our cohort, many had profound B cell depletion after rituximab or CAR‐T therapy. These children are typically heavily pretreated, having failed multiple lines of leukaemia therapy, which may alter Ig metabolism and less compensatory capacity through impaired B cell compartments, reduced plasma cell survival or disrupted neonatal Fc receptor (FcRn)‐mediated recycling. By contrast, adult SAD and PID groups are generally more balanced in their treatment histories, reducing this effect.

A second explanation is methodological. Previous models often fixed baseline IgG at 4 g L^−1^ or estimated higher values. If the true baseline is lower, fixing it too high forces the model to increase CL to match trough observations. Here, baseline IgG was estimated directly, with IgM included as a proxy for humoral capacity. The estimated baseline IgG level was 5.67 g L^−1^ for PID patients, which is consistent with values reported by Li et al. in a large cohort of PID patients aged less than 2 to 83 years.^26^ In contrast, SAD children showed roughly 50% lower baselines, in line with their profound B cell depletion. This lower baseline naturally yielded a lower apparent clearance, linking the two findings. Taken together, these results suggest that the apparently reduced clearance in SAD reflects both the biology of a heavily pretreated pediatric population and the need to estimate baseline IgG rather than fix it. Incorporating IgM as a covariate further stabilized baseline estimation and improved the biological plausibility of clearance results.

Our model incorporated estimated baseline IgG levels to account for endogenous antibody and evaluated the ability of absolute CD19+ B cell counts and IgM plasma levels to inform these baseline IgG levels. The class of antibodies produced by a B cell is determined by the heavy chain constant region gene being expressed. IgM is the first antibody produced upon activation and plays a vital role in early immune responses. Antibody class switching begins in clonally expanded antigen specific B cells from 6 days after primary activation, whereby the production of IgM is switched to other classes of antibodies, including IgG.^42^ Hence, IgM levels directly reflect B cell activity and antibody production, providing a snapshot of the functional capacity of the humoral immune system.

In contrast, absolute CD19+ B cell count measures the number of B cells but does not directly assess their functional capacity to produce specific antibodies such as IgG. While absolute CD19+ cell count is a reliable marker for identifying B cells, it does not provide information on their activation status or antibody‐producing capability. Therefore, IgM levels is a more direct and dynamic indicator of B cell function, as it reflects the immediate antibody response and the ongoing activity of the humoral immune system. Including IgM levels in the model better informs the endogenous IgG levels being produced, thereby enhancing the understanding of the effect of the therapeutic IgG being administered.

The recommended dose for SAD, ranging from 0.4 to 0.6 g L^−1^ every 4 weeks,^12^, ^16^, ^17^ maintains the therapeutic target (≥6 g L^−1^) for at least 63.8% of days during the dosing interval for the studied population (Figure 2). However, for PID patients with a higher recommended therapeutic target, only a smaller fraction (between 43.8% and 51.9%) of the simulated population reached their target at the recommended dose. Initiating therapy with a single loading dose of 1 g kg^−1^ followed by 0.4 g kg^−1^ on Day 28 increases the proportion of days above the therapeutic level to 60.7% and normalized AUC per day to 17.5 g L^−1^ day^−1^. Reducing the dosing interval from 0.4 g kg^−1^ every 28 days to 0.3 g kg^−1^ every 21 days, when combined with a loading dose of 1 g kg^−1^, further increases the likelihood of maintaining Ig levels above 8 g L^−1^ to 66.5%. This approach may accelerate the time to reach steady‐state IgG levels and reduce the overall maintenance Ig requirement. This regimen would be particularly beneficial for patients with severe clinical conditions, such as end‐organ disease or severe bronchiectasis, who may require rapid elevation of IgG plasma levels.^10^, ^16^, ^17^, ^43^ Linking plasma IgG levels with therapeutic biomarkers, such as quality of life including infection rates, would be useful in guiding future model development and dose‐efficacy assessment and thereby providing a more personalized approach to managing children with PID and SAD.

This model aimed to investigate the pharmacokinetic properties of IG in a pediatric cohort. Despite the relatively small sample size of 64 pediatric patients, we managed to capture a wide spectrum of age and body size range of the pediatric population and be statistically powered with a sufficient number of optimally timed samples to yield the unbiased and precise pharmacokinetic parameters.^44^ However, the majority of the samples collected were trough levels. While trough samples are useful for understanding steady‐state conditions and minimum drug concentrations, they do not provide a comprehensive picture of the pharmacokinetic profile, particularly for assessing peak levels and overall exposure. The inability to accurately obtain the baseline IgG levels for PID patients and variability in baseline IgG levels observed for SAD in this study (0.64 to 6.1 g L^−1^) underscores the challenge of accurately describing endogenous IgG contributions as there is no reliable method to measure endogenous IgG levels when receiving IgRT directly. Future studies with larger sample sizes, a more comprehensive sampling strategy and prospective designs are warranted to validate these findings and address the limitations identified in this analysis.

## 6 CONCLUSION

IgRT can alleviate symptomatic hypogammaglobulinaemia, thereby reducing the burden of infection risk for both PID and SAD children. The pharmacokinetic model developed in this study served as a valuable tool for clinicians managing immunoglobulin therapy in pediatric patients with PID and SAD. By individualizing the dosing strategy, this model could help optimize therapy, reduce infection risks and improve patient outcomes.

## AUTHOR CONTRIBUTIONS

Iek Leng Cheng, Joseph F. Standing, Austen Worth and Claire Booth designed the research. Iek Leng Cheng and Joseph F. Standing performed the research. Iek Leng Cheng, Zhong Hui Huang and Joseph F. Standing analysed the data. Iek Leng Cheng prepared the first draft of the manuscript. Joseph F. Standing, Austen Worth, Claire Booth and Zhong Hui Huang contributed to editing, reviewing and authorizing the final version. All authors contributed to the article and approved the submitted version.

## CONFLICT OF INTEREST STATEMENT

All authors have no conflict of interest to declare.

## Data Availability

The full‐text version of this article contains a data supplement.

## References

[1] Picard C , Al‐Herz W , Bousfiha A , et al. Primary immunodeficiency diseases: an update on the classification from the International Union of Immunological Societies Expert Committee for primary immunodeficiency 2015. J Clin Immunol. 2015;35(8):696‐726. doi:10.1007/s10875-015-0201-1 26482257 PMC4659841

[2] Tangye SG , Al‐Herz W , Bousfiha A , et al. Human inborn errors of immunity: 2019 update on the classification from the International Union of Immunological Societies Expert Committee. J Clin Immunol. 2020;40(1):24‐64. doi:10.1007/s10875-019-00737-x 31953710 PMC7082301

[3] Patel SY , Carbone J , Jolles S . The expanding field of secondary antibody deficiency: causes, diagnosis, and management. Front Immunol. 2019;10:33. doi:10.3389/fimmu.2019.00033 30800120 PMC6376447

[4] Herman KE , Tuttle KL . Overview of secondary immunodeficiency. Allergy Asthma Proc. 2024;45(5):347‐354. doi:10.2500/aap.2024.45.240063 39294908

[5] Dale RC , Brilot F , Duffy LV , et al. Utility and safety of rituximab in pediatric autoimmune and inflammatory CNS disease. Neurology. 2014;83(2):142‐150. doi:10.1212/WNL.0000000000000570 24920861 PMC4117174

[6] Pasquini MC , Hu Z‐H , Curran K , et al. Real‐world evidence of tisagenlecleucel for pediatric acute lymphoblastic leukemia and non‐Hodgkin lymphoma. Blood Adv. 2020;4(21):5414‐5424. doi:10.1182/bloodadvances.2020003092 33147337 PMC7656920

[7] Prevot J , Jolles S . Global immunoglobulin supply: steaming towards the iceberg? Curr Opin Allergy Clin Immunol. 2020;20(6):557‐564. doi:10.1097/ACI.0000000000000696 33044340 PMC7752222

[8] Justiz Vaillant AA , Qurie A . Immunodeficiency. In: StatPearls. StatPearls Publishing; 2024. 29763203

[9] Quinn J , Modell V , Orange JS , Modell F . Growth in diagnosis and treatment of primary immunodeficiency within the global Jeffrey Modell centers network. Allergy Asthma Clin Immunol. 2022;18(1):19. doi:10.1186/s13223-022-00662-6 35246253 PMC8896271

[10] Grigoriadou S , Clubbe R , Garcez T , et al. British Society for Immunology and United Kingdom Primary Immunodeficiency Network (UKPIN) consensus guideline for the management of immunoglobulin replacement therapy. Clin Exp Immunol. 2022;210(1):1‐13. doi:10.1093/cei/uxac070 35924867 PMC9585546

[11] Perez EE , Orange JS , Bonilla F , et al. Update on the use of immunoglobulin in human disease: a review of evidence. J Allergy Clin Immunol. 2017;139(3):S1‐S46. doi:10.1016/j.jaci.2016.09.023 28041678

[12] NHS England . Clinical Commissioning Policy for the Use of Therapeutic Immunoglobulin (Ig) England (2025). 2025

[13] Worch J , Makarova O , Burkhardt B . Immunreconstitution and infectious complications after rituximab treatment in children and adolescents: what do we know and what can we learn from adults? Cancer. 2015;7(1):305‐328. doi:10.3390/cancers7010305 PMC4381260 25643241

[14] Wat J , Barmettler S . Hypogammaglobulinemia after chimeric antigen receptor (CAR) T‐cell therapy: characteristics, management, and future directions. J Allergy Clin Immunol Pract. 2022;10(2):460‐466. doi:10.1016/j.jaip.2021.10.037 34757064 PMC8837681

[15] Chan EY , Ma AL , Tullus K . Hypogammaglobulinaemia following rituximab therapy in childhood nephrotic syndrome. Pediatr Nephrol. 2022;37(5):927‐931. doi:10.1007/s00467-021-05345-9 34999985

[16] Cinetto F , Francisco IE , Fenchel K , et al. Use of immunoglobulin replacement therapy in patients with secondary antibody deficiency in daily practice: a European expert Q&A‐based review. Expert Rev Hematol. 2023;16(4):237‐243. doi:10.1080/17474086.2023.2176843 37009667

[17] Otani IM , Lehman HK , Jongco AM , et al. Practical guidance for the diagnosis and Management of Secondary Hypogammaglobulinemia: a work group report of the AAAAI primary immunodeficiency and altered immune response committees. J Allergy Clin Immunol. 2022;149(5):1525‐1560. doi:10.1016/j.jaci.2022.01.025 35176351

[18] Jolles S , Michallet M , Agostini C , et al. Treating secondary antibody deficiency in patients with haematological malignancy: European expert consensus. Eur J Haematol. 2021;106(4):439‐449. doi:10.1111/ejh.13580 33453130

[19] Landersdorfer CB , Bexon M , Edelman J , et al. Pharmacokinetic modeling and simulation of biweekly subcutaneous immunoglobulin dosing in primary immunodeficiency. Postgrad Med. 2013;125(6):53‐61. doi:10.3810/pgm.2013.11.2712 24200761

[20] Tortorici MA , Lawo J‐P , Weide R , et al. Privigen® has similar pharmacokinetic properties in primary and secondary immune deficiency. Int Immunopharmacol. 2019;66:119‐126. doi:10.1016/j.intimp.2018.11.008 30447530

[21] Dumas T , Berry NS , Wolfsegger M , Jolles S , McCoy B , Yel L . Population pharmacokinetic modeling and simulation of immunoglobulin exposure with varying dosing intervals of subcutaneous immunoglobulin 20% (Ig20Gly) in patients with primary immunodeficiency diseases. Int Immunopharmacol. 2019;71:404‐410. doi:10.1016/j.intimp.2019.03.034 30952104

[22] Zhang Y , Baheti G , Chapdelaine H , et al. Population pharmacokinetic analysis of weekly and biweekly IgPro20 (Hizentra®) dosing in patients with primary immunodeficiency. Int Immunopharmacol. 2020;81:106005. doi:10.1016/j.intimp.2019.106005 31806567

[23] Luo D , Baheti G , Tortorici MA , Hofmann J , Rojavin MA . Pharmacometric analysis of IgPro10 in Japanese and non‐Japanese patients with primary immunodeficiency. Clin Ther. 2020;42(1):196‐209.e5. doi:10.1016/j.clinthera.2019.11.013 31910997

[24] Tegenge MA , Mahmood I . Population pharmacokinetics of immunoglobulin intravenous preparation in very low birth weight neonates. Int Immunopharmacol. 2020;80:106192. doi:10.1016/j.intimp.2020.106192 31931361

[25] Lee JL , Mohd Saffian S , Makmor‐Bakry M , et al. Population pharmacokinetic modelling of intravenous immunoglobulin in patients with predominantly antibody deficiencies. Br J Clin Pharmacol. 2021;87(7):2956‐2966. doi:10.1111/bcp.14712 33377197

[26] Li Z , Follman K , Freshwater E , Engler F , Yel L . Integrated population pharmacokinetics of immunoglobulin G following intravenous or subcutaneous administration of various immunoglobulin products in patients with primary immunodeficiencies. Int Immunopharmacol. 2022;113(Pt A):109331. doi:10.1016/j.intimp.2022.109331 36461591

[27] Navarro‐Mora G , Alberti JJ , Mondou E , et al. Pharmacokinetic modeling and simulation of subcutaneous and intravenous IgG dosing in patients with primary immunodeficiency diseases. Int Immunopharmacol. 2022;104:108472. doi:10.1016/j.intimp.2021.108472 35008008

[28] Lee JL , Mohamed Shah N , Makmor‐Bakry M , Islahudin F , Alias H , Mohd Saffian S . Population pharmacokinetic model of intravenous immunoglobulin in patients treated for various immune system disorders. Clin Ther. 2024;46(12):e25‐e37. doi:10.1016/j.clinthera.2024.09.018 39366801

[29] Fokkink WJR , van Tilburg SJ , de Winter BCM , et al. Population pharmacokinetic modelling of intravenous immunoglobulin treatment in patients with Guillain–Barré syndrome. Clin Pharmacokinet. 2022;61(9):1285‐1296. doi:10.1007/s40262-022-01136-z 35781631 PMC9439991

[30] Wang K , Wei G , Liu D . CD19: a biomarker for B cell development, lymphoma diagnosis and therapy. Exp Hematol Oncol. 2012;1(1):36. doi:10.1186/2162-3619-1-36 23210908 PMC3520838

[31] Burrell CJ , Howard CR , Murphy FA . Chapter 6—adaptive immune responses to infection. In: Burrell CJ , Howard CR , Murphy FA , eds. Fenner and White's Medical Virology. 5th ed. Academic Press; 2017:65‐76. doi:10.1016/B978-0-12-375156-0.00006-0

[32] Aydogan M , Eifan A , Gocmen I , Ozdemir C , Bahceciler N , Barlan I . Clinical and immunologic features of pediatric patients with common variable immunodeficiency and respiratory complications. J Invest Allergol Clin Immunol. 2008;18:260‐265. 18714533

[33] Germovsek E , Barker CIS , Sharland M , Standing JF . Scaling clearance in paediatric pharmacokinetics: all models are wrong, which are useful? Br J Clin Pharmacol. 2017;83(4):777‐790. doi:10.1111/bcp.13160 27767204 PMC5346879

[34] Alexander SPH , Christopoulos A , Davenport AP , et al. The concise guide to PHARMACOLOGY 2021/22: catalytic receptors. Br J Pharmacol. 2021;178(S1):S264‐S312. doi:10.1111/bph.15539 34529829

[35] Alexander SPH , Christopoulos A , Davenport AP , et al. The concise guide to PHARMACOLOGY 2021/22: other protein targets. Br J Pharmacol. 2021;178(S1):S431‐S514. doi:10.1111/bph.15541 PMC9513948 34529830

[36] Walker JC , Smolders M a JC , Gemen EFA , Antonius T a J , Leuvenink J , De Vries E . Development of lymphocyte subpopulations in preterm infants. Scand J Immunol. 2011;73(1):53‐58. doi:10.1111/j.1365-3083.2010.02473.x 21129003

[37] Zemlin M , Hoersch G , Zemlin C , et al. The postnatal maturation of the immunoglobulin heavy chain IgG repertoire in human preterm neonates is slower than in term neonates1. J Immunol. 2007;178(2):1180‐1188. doi:10.4049/jimmunol.178.2.1180 17202383

[38] González‐Sales M , Holford N , Bonnefois G , Desrochers J . Wide size dispersion and use of body composition and maturation improves the reliability of allometric exponent estimates. J Pharmacokinet Pharmacodyn. 2022;49(2):151‐165. doi:10.1007/s10928-021-09788-3 34609707

[39] Calvier EAM , Krekels EHJ , Välitalo PAJ , et al. Allometric scaling of clearance in paediatric patients: when does the magic of 0.75 fade? Clin Pharmacokinet. 2017;56(3):273‐285. doi:10.1007/s40262-016-0436-x 27510367 PMC5315734

[40] van Valkengoed DW , Krekels EHJ , Knibbe CAJ . All you need to know about allometric scaling: an integrative review on the theoretical basis, empirical evidence, and application in human pharmacology. Clin Pharmacokinet. 2025;64(2):173‐192. doi:10.1007/s40262-024-01444-6 39644458 PMC11782306

[41] Song D , Prahl M , Gaw SL , et al. Passive and active immunity in infants born to mothers with SARS‐CoV‐2 infection during pregnancy: prospective cohort study. BMJ Open. 2021;11(7):e053036. doi:10.1136/bmjopen-2021-053036 PMC8264915 34234001

[42] Stavnezer J . Immunoglobulin class switching. Curr Opin Immunol. 1996;8(2):199‐205. doi:10.1016/S0952-7915(96)80058-6 8725943

[43] Shillitoe B , Hollingsworth R , Foster M , et al. Immunoglobulin use in immune deficiency in the UK: a report of the UKPID and National Immunoglobulin Databases. Clin Med. 2018;18(5):364‐370. doi:10.7861/clinmedicine.18-5-364 PMC6334102 30287427

[44] Ribbing J , Niclas Jonsson E . Power, selection bias and predictive performance of the population pharmacokinetic covariate model. J Pharmacokinet Pharmacodyn. 2004;31(2):109‐134. doi:10.1023/B:JOPA.0000034404.86036.72 15379381

